# Efficacy of Continuous Saline Irrigation Therapy for Descending Necrotizing Mediastinitis

**DOI:** 10.1055/s-0043-1775559

**Published:** 2023-09-28

**Authors:** Takuya Ohashi, Mitsumasa Kawago, Yoshimitsu Hirai, Yumi Yata, Aya Fusamoto, Hideto Iguchi, Takahito Nakaya, Megumi Kiyoi, Miwako Miyasaka, Mari Kawaji, Yuki Fujiwara, Yoshiharu Nishimura

**Affiliations:** 1 Department of Thoracic and Cardiovascular Surgery, Wakayama Medical University, Wakayama, Japan

**Keywords:** descending necrotizing mediastinitis, mediastinal drainage, irrigation therapy

## Abstract

**Objectives** Descending necrotizing mediastinitis (DNM) is a poor prognosis disease. This study aims to examine the patient background and treatment of DNM and to identify more effective treatments for DNM.

**Methods** The patient background and treatment of 11 patients who underwent surgery for DNM between November 2010 and June 2021 were studied. The patients were divided into six patients who underwent continuous saline irrigation (group I) and five patients who did not (group N). The differences in the drainage duration and length of hospital stay between the two groups were retrospectively investigated.

**Results** Eleven patients were treated for DNM: six male and five female, with a median age of 61 years (35–79). Comorbidities included diabetes mellitus in three cases; one patient was administered steroids. The pathways of occurrence were anterior tracheal gap/vascular visceral gap/posterior visceral gap in group I (2/1/2) and group N (0/2/4). Progression was I/IIA/IIB according to Endo's classification in group I (1/1/4) and group N (3/1/1). The mean duration of irrigation was 9.0 ± 3.7 days, and the drainage duration in group I was 17.5 ± 8.2 days, which was significantly shorter than 31 ± 13.6 days in group N ( *p* < 0.048). The hospital stays in group I was 29.3 ± 8.4 days, which was significantly shorter than that in group N (68 ± 27.1 days; *p* < 0.015).

**Conclusions** Irrigation therapy significantly shortened the drainage duration and hospital stay. Irrigation is a useful treatment for DNM.

Descending necrotizing mediastinitis (DNM) is a serious condition caused by deep cervical infections, odontogenic infections, or Ludwig's angina 1 that spread to the mediastinum. DNM is a rapidly worsening condition and requires prompt treatment, which consists of antibiotic therapy and drainage of the cervical and mediastinal areas where the infection has spread. Due to the low incidence of DNM, there are few published reports 2 3 4 and no guidelines for its treatment. However, due to the high mortality rate of 25 to 40%, 2 appropriate treatment options need to be considered. In this study, we have retrospectively reviewed our experience with DNM. We also examined whether the treatment progress, such as the duration of drainage and hospital stay, differed with and without continuous saline irrigation therapy.

## Patients and Methods

This study was approved by the Wakayama Medical University Medical Research Ethics Committee (3320) on October 28, 2021, and all patients consented to this study by opt-out. Patient inclusion criteria were all patients with DNM. Exclusion criteria were DNM patients who died of other diseases. This study was based on the criteria of the STROBE Statement. Among the 12 patients diagnosed with DNM in our department between November 2010 and February 2022, 11 patients (15 surgical cases) were studied, excluding one patient who died of another disease. Depending on the extent of extension, DNM were classified into three groups using the ENDO classification. 5 The localized type (type I) is confined from the superior mediastinum cephalad to the tracheal bifurcation, while the extensive type extends to the caudal mediastinum. In the extensive type, the anterior mediastinal type (type IIA) is limited to the mainly anterior mediastinal; the posterior mediastinal type (type IIB) extends through the esophageal interstitium to the inferior mediastinum. We examined the underlying disease, the route of spread using the ENDO classification, the causative organism, the surgical drainage methods, drainage duration, and the length of hospital stay. As for surgical drainage methods, mediastinal drainage by cervical approach was mandatory. Thoracic drainage was mandatory if the mediastinum could not be drained via cervical approach or if the pleural fluid showed bacteria on Gram stain. Additional surgical drainage methods were determined by the attending physician depending on the degree of abscess development.

Furthermore, 11 patients who underwent surgery with DNM were divided into two groups: six patients who underwent continuous saline irrigation after the surgery (irrigation group: group I) and five patients who did not undergo irrigation (nonirrigation group: group N). In the continuous saline irrigation group, the wounds were irrigated with 1,000 to 2,000 mL of saline solution per day beginning immediately after the surgery. When the attending physician judged that the wound had not been irrigated sufficiently, additional irrigation with 100 to 500 mL of saline was performed each time. Saline irrigation was continued until culture tests were negative. Saline irrigation was performed at the attending physician's discretion.

### Statistical Analysis

Statistical analysis was performed using JMP version 14.0. The *t* -test was used for bivariate comparisons following a normal distribution, and the Mann–Whitney U test was used for bivariate comparisons not following a normal distribution. *p* -Values of less than or equal to 0.05 were considered significantly different.

## Results

The details of the 11 patients who underwent surgery for DNM are shown in Table 1 .

**Table 1:** Characteristics of all DNM cases

Median age		68 (35–79)
Sex	Men	6
	Female	5
Medical history	Diabetes mellitus	3
	Steroid administration	1
Inflammatory transmission pathway	Pretracheal space	2
	Vascular visceral space	1
	Posterior visceral space	8
ENDO classification	Type I	2
	Type IIA	3
	Type IIB	6
Microbial investigations	Streptococcus constellatus	3
	Streptococcus agalactiae	1
	Streptococcus milleri	1
	Streptococcus sanguis	1
	Stenotrophomonas maltophilia	1
	Staphylococcus epidermis	1
	Streptococcus species	1
Pleural effusion	Yes	7
	No	4

In 10 out of 11 patients, cervical and mediastinal drainage was performed on the second day after admission. The remaining patient, who initially had only a neck abscess, underwent cervical drainage on the day of admission but later underwent mediastinal drainage on the fourth day after admission as the abscess had spread to the mediastinum ( Table 2 ).

**Table 2:** Surgery and postoperative course of all DNM cases

Time from diagnosis of DNM to surgical cervical drainage (days)		1.2 (1–2)
Time from diagnosis of DNM to surgical mediastinal drainage (days)		1.5 (1–4)
Initial mediastinal drainage methods	Cervical approach	8
	Cervical and thoracic approaches	2
	Cervical, thoracic, and orthopaedic posterior cervical decompression	1
Additional mediastinal drainage methods	Cervical approach	1
	Thoracic approach	2
	Chamberlain approach	1
Number of mediastinal drains per patient		3.1 (1–7)
Mediastinal drainage duration (days)		23.6 (10–48)
Hospital stays (days)		46.9 (18–104)

Abbreviation: DNM, descending necrotizing mediastinitis.

Initial mediastinal drainage was performed by cervical approach in eight patients, cervical and thoracic approach in two patients, and cervical, thoracic, and orthopaedic posterior cervical decompression in one patient.

Additional mediastinal drainage methods included: in two cases, the thoracic approach was added to the cervical approach; in one case, the cervical approach was added again to the cervical approach; and in one case, the chamberlain approach was added to the cervical and thoracic approaches.

The mean number of drains inserted per operation was 3.1 ± 1.1 The average duration of drainage was 23.6 days, and the average hospital stay was 46.9 days. No DNM-related deaths occurred postoperatively.

We examined group I (six cases), in which saline irrigation was performed, and group N (five cases), in which saline irrigation was not performed ( Table 3 ).

**Table 3:** Characteristics compared with group I and group N

		Group I	Group N
Median age		65.5 (35–79)	69 (58–73)
Sex	Men	2	3
	Female	4	2
Medical history	Diabetes mellitus	2	1
	Steroid administration	1	0
Inflammatory transmission pathway	Pretracheal space	2	0
	Vascular visceral space	1	2
	Posterior visceral space	2	4
ENDO classification	Type I	1	3
	Type IIA	1	1
	Type IIB	4	1
Mean irrigation period (days)		9.0 ± 3.7	
Mediastinal drainage duration (days)		17.5 ± 8.2	31 ± 13.6
Hospital stays (days)		29.3 ± 8.4	68 ± 27.1

In group I, there was one case of type I, one case of type IIA, and four cases of type IIB according to the ENDO classification. In group N, there were three cases of type I, one case of type IIA, and one case of type IIB.

**Fig. 1:**
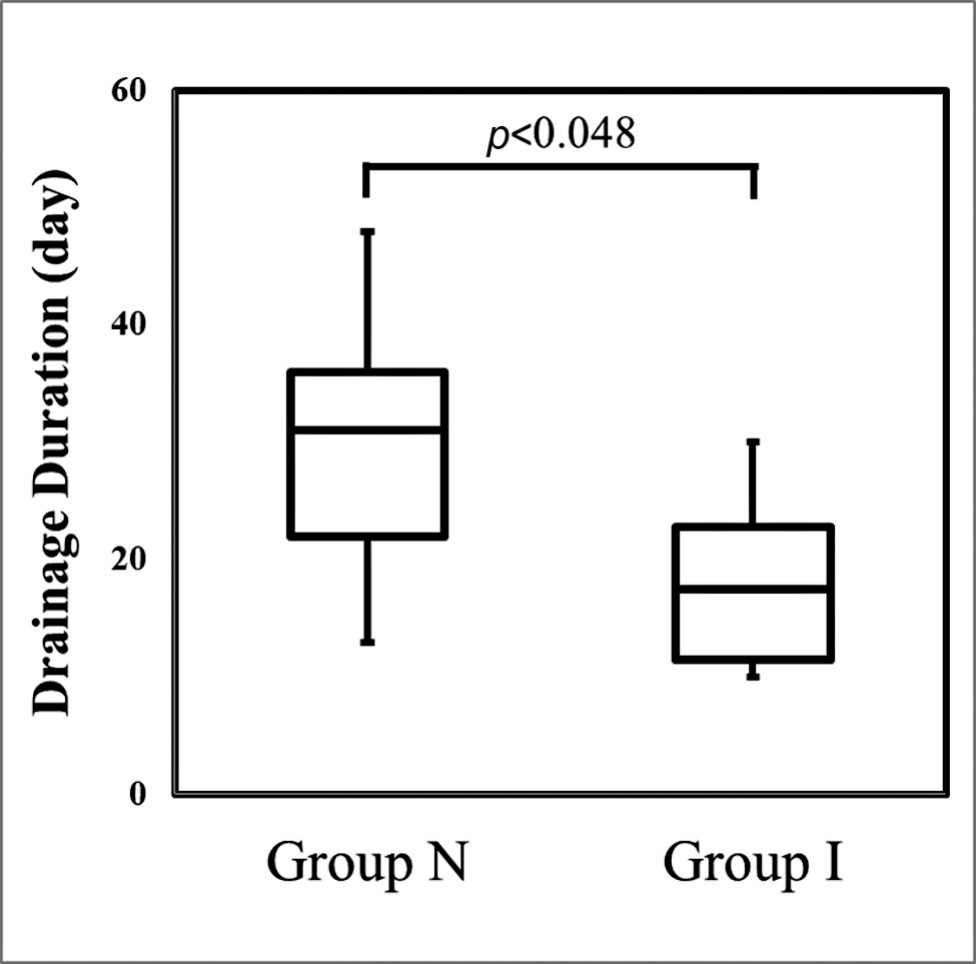
Differences in drainage duration between group I and group N.

**Fig. 2:**
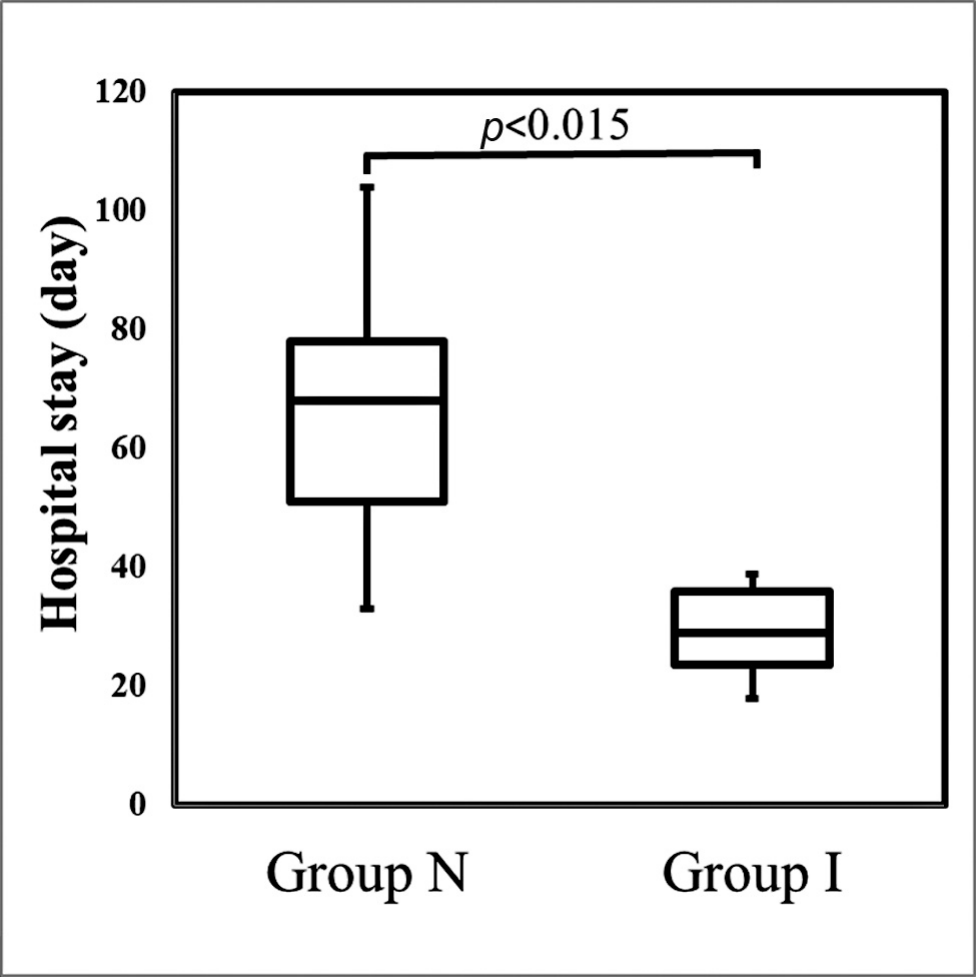
Differences in hospital stay between group I and group N.

The drainage period was 17.5 ± 8.2 days in group I, significantly shorter than 31 ± 13.6 days in group N ( *p* < 0.048; Fig. 1 ). The hospital stay was also significantly shorter in group I (29.3 ± 8.4 days) compared with that of group N (68 ± 27.1 days; *p* < 0.015; Fig. 2 . The mean duration of saline irrigation in group I was 9 ± 3.7 days.

## Discussion

DNM is caused by a deep cervical infection or odontogenic infection that spreads to the mediastinum through the intervascular space and fascial space.

The mainstay of treatment is antibiotic therapy and drainage. Drainage mostly requires procedures to be performed under general anesthesia, with additional drainage of the thoracic and mediastinal regions in addition to the cervical region.

The presence of diabetes mellitus as an underlying disease exacerbates the risk of deep neck infections and odontogenic infections progressing to DNM. 3 The high mortality rate requires prompt and individualized treatment for each patient. 6 Furthermore, there are reports that diabetes was a poor prognostic factor for DNM. 4 In a report by Roccia et al, 7 six out of 23 (26%) patients with DNM had underlying diabetes mellitus. In our case, three out of 11 patients (27%) had diabetes mellitus, which is similar to that reported in previous reports.

A systematic review of 26 articles 8 reported that among 480 patients with DNM, 189 (39%) had type I, and 249 (52%) were reported to have type IIA + IIB. The proportion of patients with DNM in our hospital was generally similar; four patients had type I (33%) and eight patients had type IIA + IIB (67%).

The most common drainage method for DNM is cervical and additional mediastinal drainage. 5 Cervical drainage alone resulted in a mortality rate of 47%, which was reduced to 19% when combined with mediastinal drainage. 9

However, there are various approaches for mediastinal drainage, including posterolateral incision, sternotomy, glenoid incision, and clamshell incision. Furthermore, there are differences in technique, such as open surgery or thoracoscopic surgery and the use of a mediastinoscope. Endo et al stated that type I requires a cervical approach, type IIA requires a subxiphoid approach in addition to a cervical approach, and type IIB requires a transthoracic approach in addition to a cervical approach for adequate mediastinal drainage surgery. However, Hsu et al reported that there was no difference in the length of hospital stay and mortality between mediastinal drainage by cervical approach alone and by combined cervical and thoracic approach. 10 Although mediastinal drainage is essential, a standardized approach has not yet been established.

Anatomically, the mediastinum and thoracic cavity are bordered by the mural pleura, a membrane structure that prevents the spread of infection. In this study, none of the seven cases in which pleural fluid could be obtained showed bacteria in the pleural fluid culture; in four out of five type IIB cases, the bacterium was identified in the mediastinal fluid. However, the bacterium was not identified in the pleural cavity fluid in any of the cases.

It is important not to hesitate to perform transthoracic drainage, including open thoracic drainage, if there is widespread inflammation in the mediastinum and thoracic cavity or if mediastinal drainage by a transcervical approach is not sufficient. 11

The inability to identify an inflammatory organism from within the thoracic cavity suggests that thoracic drainage may not be essential if mediastinal drainage can be adequately achieved via a cervical approach. Rather, the cervical approach alone may be more advantageous as it allows localization of the infection only in the mediastinum.

Several papers have reported on the effectiveness of saline irrigation, but most are case reports. 12 13 14 15 Iwata et al 16 described the cases of 10 patients with DNM who were irrigated with 1,000 to 2,000 mL saline twice daily and had a good course with an average drainage period of 26.7 ± 17.0 days and an average hospital stay of 62.3 ± 33.9 days.

No previous reports of saline irrigation have compared saline irrigation with no saline irrigation. In contrast, this study is a comparison of the saline irrigation group (group I) and the nonsaline irrigation group (group N) that revealed a predominantly shorter drainage period and hospital stay.

Furthermore, group I consisted of more type IIB cases. As irrigation was performed at the discretion of the attending physician, it is noteworthy that group I contained more severe cases with more extensive abscess cavities. Nevertheless, group I had a shorter drainage period and hospital stay.

Our study was comprehensive, with no deaths occurring due to DNM, although one patient died due to cancer.

### Limitation

Several limitations of this study exist. First, the study did not randomize patients due to the small number of cases. Second, this is a backward-looking study, and the presence of selection bias by the attending physician and many other biases that we cannot address cannot be ruled out. This study has several limitations, and the results may need to be interpreted with caution. Large-scale prospective double-blind studies are desirable but may be difficult because the disease is not highly prevalent.

## Conclusion

Saline irrigation after drainage significantly reduced the drainage period and the length of hospital stay. Thus, saline irrigation is a useful treatment modality for DNM.
